# Criminal Lunatics

**Published:** 1881-09

**Authors:** 


### CRIMINAL LUNATICS.

The following, which travels back from England, like the Americo-English Stiltan cheese, in the columns of the *Medical Times and Gazette,* is not inappropriate at the present moment or to this issue of the *Journal:*

"A strong feeling seems to be growing up in the United States against the practice almost universally prevalent there, and which, indeed, still prevails to a considerable extent in this country, of mingling the criminal with the non-criminal insane in the wards of lunatic asylums. This feeling finds vigorous expression in the Report of the Managers of the New Jersy State Asylum, which has just been transmitted to England. With a force and freedom of language unusual in formal official documents in Europe, they inveige against the objectionable arrangement. 'To place insane convicts,' they say, 'in the same institution and the same appartments with insane patients from the families of our best citizens is inconsistent with every dictate of propriety. The very proposition excites a feeling of revulsion in every bosom· Think of the children of our late Governor (honored as he was while living) thrown together in the same section of the asylum with convicts from the State prison! A voice from the tomb of the distinguished dead cries out in condemnation of such an arrangement, And the voice of the living re-echoes the sentiment, and cries out against the law that requires it. Who is responsible for this? The superintendent does the best he can under the law. The Board of Managers have again and again called attention to it and urged a remedy. Governor Parker called the attention of the legislature to it in his message in 1875. Governor Bell, in equally strong terms, presented the matter in his message of 1877. The Senate passed a bill providing a remedy, but it slumbered in the lap of the committee of the House. The evil continues and increases. Let it be removed. Cannot a wing for insane convicts be provided in connection with the state prison? If this cannot be done, a separate building sufficiently secure and suitable for the purpose should be erected for their accommodation. The problem is one that must be solved sooner or later. Let it be done without delay. In our judgment the purity of the institution demands it. Public policy demands it. The ends of justice require it. The good name of the State calls for it. The legislature that meets the case and provides a proper remedy will receive the grateful acknowledgements of the entire constituency of the State of New Jersey.' The medical superintendent of the asylum follows in the wake of the board of managers, and in somewhat calmer accents and more argumentative strain enforces the same theme. He points out that an ordinary lunatic hospital is not constructed in such a manner as to render it suitable for the detention of persons who add determined criminal propensities to insanity, and that it would destroy its hospital character to adapt it to that class, and he pleads for the erection of a separate asylum, meeting the requirements of security, and so organized as to supply humane treatment to the really insane without creating any incentive to convicts to feign insanity. We commend his observations, as well as the more oratorical and uncompromising remarks of the board of managers, to the consideration of that mixed departmental and parliamentary committee which has been deliberating for at least twelve months on all questions bearing on criminal lunacy in this country. If we might adopt the figure of the New Jersey board of managers, we should say that they have allowed the subject to slumber in their lap, and that their report is awaited, if not with impatience, yet with a conviction that it is slightly overdue. The questions involved in the reference to the committee are doubtless somewhat complex and difficult, but the information which is to serve as the basis of the answers returned to them ought to be readily accessible.

